# Human anti-rabies care in the State of São Paulo: evaluating prophylaxis conduct in individuals attacked by dogs and cats

**DOI:** 10.1590/0037-8682-0170-2023

**Published:** 2024-10-28

**Authors:** Bruno Fonseca Martins da Costa Andrade, Luzia Helena Queiroz, Márcia Marinho

**Affiliations:** 1 Universidade Estadual Paulista “Júlio de Mesquita Filho”, Faculdade de Medicina Veterinária de Araçatuba, Programa de Pós-Graduação em Saúde Animal, Araçatuba, SP, Brasil.; 2 Universidade Estadual Paulista “Júlio de Mesquita Filho”, Faculdade de Medicina Veterinária de Araçatuba, Departamento de Produção e Saúde Animal, Araçatuba, SP, Brasil.

**Keywords:** Rabies, Immunization schedules, Rabies vaccines, Post-exposure prophylaxis, Human Bites

## Abstract

**Background:**

Rabies is a zoonosis usually transmitted to mammals via contact between the saliva of infected animals and either the skin or mucosa of the attacked individual, and post-exposure prophylaxis (PEP) is the only way to avoid the disease. This study aimed to perform a descriptive analysis of PEP after attacks by dogs and cats in the state of São Paulo.

**Methods:**

We analyzed the disease compulsory notification forms Human Anti-Rabies Care (CID10: W64), available in the Notifiable Diseases Information System (SINAN), from 2013 to 2017. Treatment adequacy was analyzed according to the parameters established by the Brazilian Health Ministry.

**Results:**

A total of 572,889 notifications were reported during the study period, 94.1% (538,975) of which corresponded to dog or cat attacks, with an occurrence of 26.9 cases per 10,000 inhabitants. Among the recommended procedures, the most frequent was the observation of the animals for 10 days (44.4%), which was adopted inappropriately at a lower frequency. Prophylactic conduct was adequate in 68.8% of the cases, but only 55.5% of the individuals received adequate treatment. More than 112 thousand individuals (31,4%) received a correct recommendation for PEP but did not receive adequate treatment, leading to 246,787 doses of the vaccine and 8,888 doses of rabies immunoglobulin administered without following the recommendations of the Ministry of Health.

**Conclusions:**

The use of immunobiologicals is excessive, indicating the need for investment in training health professionals to follow the recommendations of the Ministry of Health.

## INTRODUCTION

Rabies is an acute encephalomyelitis caused by infection with a virus belonging to the *Lyssavirus* genus of the Rhabdoviridae family. More than 99% of all human rabies-related deaths worldwide occur in Asia and Africa as a result of bites from infected dogs^1^. Although there are treatments using the Milwaukee Protocol and the Recife Protocol^2^ ^,^ ^3^, the diagnosis of rabies is statistically synonymous with death, and once clinical signs appear, there is no proven treatment for a total cure^1^ ^,^ ^4^ ^,^ ^5^. 

The urban cycle is the most important for keeping human rabies alive worldwide, as dogs are still the main reservoir and source of viral infection, being the species with the greatest epidemiological relevance^6^. Canine rabies, caused by antigenic variants 1 and 2 (AgV1 and AgV2), is directly transmitted between dogs and was probably introduced to the New World during colonization^7^. Brazil has made substantial progress towards the elimination of dog-mediated rabies in recent decades; however, some cases persist in the northeastern states, and a major outbreak has occurred in recent years in municipalities in the state of Mato Grosso do Sul, close to the border with Bolivia^6^ ^,^ ^8^. Some South American countries (Chile, Uruguay, and part of Argentina) and Southern Brazil, including São Paulo (SP) and Rio de Janeiro (RJ), are free of dog rabies^9^. 

From 2009 to 2013, almost three million treatments with anti-rabies post-exposure prophylaxis (PEP) were registered in the country^10^. This number increased to almost four million between 2014 and 2019^11^. Most instances of PEP occurred after dog and cat attacks, which were recorded in Brazil’s Southeast and Northeast regions^10^ ^,^ ^11^ and São Paulo had the highest number of human anti-rabies post-exposure prophylaxis notifications^11^. Between 2000 and 2017, 188 human rabies cases were diagnosed in Brazil caused by different species, and 45.7% of the rabies cases were caused by dog attacks, most frequently by dog bites (81.9%)^12^. The last human dog-mediated rabies case in SP was reported in 1995 at Ribeirão Preto and in 1997 at Avanhandava^13^.

Human rabies can be prevented by the appropriate use of pre- and post-exposure prophylaxis. The first (pre-exposure) is recommended for any individual who is at a continual, frequent, or increased risk of exposure to the rabies virus as a result of their occupation/profession or residence, whereas PEP is indicated when there is a risk of infection as a result of exposure to the virus^14^. PEP anti-rabies care is a mandatory notifiable event, so that the risk of exposure to the virus is assessed using the information provided by the patient when filling out the specific form^3^. 

In Brazil, the post-exposure approach must be adopted following the recommendations of the World Health Organization (WHO),^15^ which are the basis for the Technical Standards for Rabies Prophylaxis of the Ministry of Health (Ministério da Saúde, MS)^16^. Several factors determine the choice of the most appropriate conduct, especially the aggressor species, its health status at the time of aggression, and the nature of the exposure. For minor injuries and those caused via indirect contact only, the recommendation is to observe the animal for 10 days, and the individual is released from rabies prophylaxis, requiring the use of immunobiologicals (vaccine and serum)^16^.

Studies performed in Brazil have shown that, in some cases, the recommended treatment approaches do not adequately follow the guidelines of the MS regarding disease prophylaxis. Every time adequate treatments are not recommended or used, there is either a risk of infection by the rabies virus or a waste of immunobiologicals when administering the vaccine or serum is unnecessary or excessive^17^ ^-^ ^19^. 

Research conducted in Araçatuba, northwest of the state of SP, evaluated the conduct adopted for rabies prophylaxis in two different periods: uncontrolled-with dog rabies cases (1990-1996) and controlled-no dog rabies cases (1997-2010). That study showed that the conduct was more adequate in the first period than the second, but in both periods, the serum and vaccine PEP recommendations were excessive according to the technical recommendations of the MS^14^. In another study, considering the total number of PEP treatments in all Brazilian states from 2014 to 2019, prophylactic conduct was classified as adequate in 57.8% of the notifications and inadequate in 42.2%^11^. In the state of SP, in many cases of attacks by different animal species, the recommended conduct does not follow the official guidance of the MS, especially animal observation^19^.

Based on the results already observed in Brazil and the northwestern regions of the state of SP, and considering the high number of notifications of human anti-rabies care attendance annually registered in the Notifiable Diseases Information System (SINAN)^20^, as well as the epidemiological situation of controlled rabies in the state, this study aimed to evaluate the conduct adopted in human anti-rabies care attendance after aggression from dogs and cats only, in all regions of the state of SP, from 2013 to 2017.

## METHODS

This descriptive study analyzed the disease compulsory notification forms Human Anti-Rabies Care (CID10-W64) available in the Notifiable Diseases Information System (SINAN)^20^, registered from 2013 to 2017. The state of SP was selected because it had the highest number of post-exposure care notifications in the whole country between 2009 and 2013^10^ and 2014 and 2019^11^, based on previous studies^14^.

### • Ethical Considerations

The data stored in the SINAN platform were provided by the Pasteur Institute of São Paulo, after approval by the Research Ethics Committee (CAAE n° 73202317.2.0000.5420) of the Faculty of Dentistry of the Universidade Estadual Paulista "Júlio de Mesquita Filho"/UNESP, in Araçatuba, SP.

The available data, except for variables regarding individual identification data, were tabulated in Microsoft Excel spreadsheets. The Annual Percent Change (APC) of medical attendance was obtained by the statistical method of log-linear regression and Poisson distribution using the Joinpoint Regression Program (version 4.8.0.1) available at http://surveillance.cancer.gov/joinpoint/. Spatial analysis was performed using ArqGis 10.8 software, while other statistical analyses were performed in the R^21^ environment using a 95% confidence interval (CI).

All notified antirabies care related to accidents caused by dogs and cats only that occurred in municipalities in the state of SP were included in the study. Accidents caused by any other species were excluded.

São Paulo is divided into 17 Regional Health Departments (RHD), with varying numbers of cities and inhabitants. Therefore, the population density was obtained for the period evaluated using the estimated human population published by the Brazilian Institute of Geography and Statistics (IBGE)^22^

The type of exposure was classified as: (1) *Indirect contact,* when neither injuries nor contact with the mucosa was reported; (2) *Minor accident,* when superficial injuries, usually single, were reported on the torso and limbs (except for the hands, digital pulps, and soles of the feet); (3) *Severe accident,* when multiple or severe injuries were observed in the head, hands/feet, or licking of mucous membranes; and (4) *Inconclusive,* when data on injury location, type, and characteristics were missing or incomplete^16^

The assessment of the performed treatment adequacy was based on the technical standards for rabies prophylaxis of the MS^5^ ^,^ ^16^ by analyzing two measures: (1) the *prescribed or recommended conduct*, which considered the type of exposure and the clinical situation of the animal at the time of aggression, and (2) the *prophylactic conduct or performed treatment* such as prophylaxis or release from treatment, according to the type of exposure, as well as the animal’s clinical condition at the time of aggression and after the observation period.

As the notification form has no fields intended to assess the animal origin (from an area of controlled or uncontrolled rabies) and lifestyle habits (domiciled or not domiciled), as described in the technical standards for rabies prophylaxis^16^, we considered that the animal situation was favorable for indicating the 10-day observation period. Among the reports recommending pre-exposure and/or re-exposure treatments, only those with previous pre- or post-exposure prophylaxis records were considered eligible. The notification form has no specific field to inform whether pre-exposure prophylaxis had confirmed serology (titration), so all were considered as having a titer greater than or equal to 0.5 UI/ml, while for post-exposure and individuals with previous post-treatment it was considered that it was carried out completely.

Prophylactic conduct was classified as inadequate when the use of vaccine or serum was prescribed inappropriately for the type of exposure or when the number of doses indicated was not compatible with the treatments recommended by the MS^16^. Administering 4 doses of the vaccine to replace the 5-dose schedule was considered adequate treatment for occurrences as of August 2017, according to Information Note No. 26/2017 of the Ministry of Health^23^.

## RESULTS AND DISCUSSION

During the five years of this study, 572,889 human anti-rabies care forms were completed in the state of SP, with 538,975 (94.1%) occurrences caused by either dogs or cats, averaging 107,795 cases per year, 92.7% of which were in urban areas. The annual average occurrence was also higher than that in other states, such as Minas Gerais^24^, Paraná^25^ and Ceará^26^. 

**FIGURE 1:**
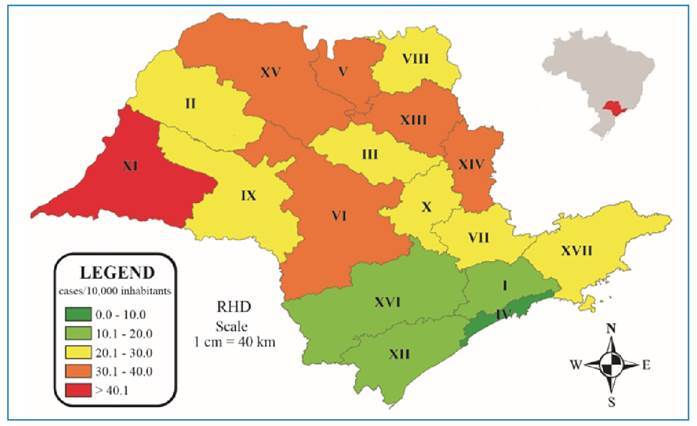
Incidence of notified human anti-rabies care per 10,000 inhabitants per Regional Health Department (RHD I to XVII) in the state of São Paulo, Brazil, from 2013 to 2017. Source: Author, 2024.

The incidence coefficient (number of cases/10,000 inhabitants) of anti-rabies care during the analyzed period varied among the RHD in the state (Figure 1). Nevertheless, there was no significant difference when the incidence by RHD was analyzed over time (APC=1.8%; 95% CI: -1.3 to 5.1; p=0.228). The median for the entire state was 26.9 cases per 10,000 inhabitants; the lowest rate was observed in RHD IV, Baixada Santista (14.6/10,000 inhabitants), and the highest in RHD XI, Presidente Prudente (42.6 /10,000 inhabitants). In Brazil, between 2008 and 2016, an incidence coefficient higher than 20 anti-rabies care attendance/10,000 inhabitants was recorded in 88.9% of the states for individuals bitten by dogs^18^.

The aggressions reported were predominantly caused by the canine species (88.7%; 477,834/538,975) and the animal clinical status was considered “healthy” in 71.6% of the cases at the time of the aggression, followed by dead/missing (13%), suspect (12.8%), and rabid status (0.2%); in 2.4% of cases there was no information on health status. Animal observations were possible in 72.8% (392,611/538,975) of the cases; after the recommended observation period, the animals remained healthy in 55.5% of the cases, followed by ignored health status (17.6%), dead/euthanasia (5%), and rabid (0.2%), and in 21.7% of the cases, the health status was not reported. Dogs are recognized as the main aggressors in urban rabies; in most cases, after the observation period, the clinical state at the time of aggression has also been reported as “healthy” in several other studies^14^ ^,^ ^24^ ^-^ ^26^. 

A total of 21.7% (117,013/538,975) of the notified cases had no information on the animal’s clinical status after the observation period. A lack of information on this issue was also frequent and high in studies conducted in other Brazilian states, such as Pernambuco (58.3%)^27^ and Paraíba (73%)^28^. This result suggests negligence or lack of training in filling out the care forms as well as inadequate follow-up of cases to record the treatment outcomes of each individual. The final situation of the aggressor is extremely relevant as it allows for changing the adopted prophylactic conduct as needed by either starting the vaccination schedule or suspending the use of vaccine doses to be administered^5^ ^,^ ^16^.

The most frequently recommended/prescribed conduct in the state of SP (Table 1) was animal observation only for 10 days (44.4%); for the rest of Brazil, 50.4% of the recommended conduct between 2009 and 2013 was animal observation plus its vaccination^29^. A study involving all the Brazilian states from 2014 to 2019 also reported a high percentage of observation of the animal + vaccine (44.2%) adopted, in contrast to animal observation only (26.6%)^11^. In the state of SP, it is recommended that, in patients attacked by animals subject to observation and without clinical signs suggestive of rabies, treatment must not be indicated^30^. 

**TABLE 1:** Distribution of conduct prescribed and anti-rabies treatment performed after PEP visits in the state of São Paulo, Brazil, from 2013 to 2017.

Characteristic	Occurrences	Percent
**Prescribed conduct**	**538,975**	**100.0**
Pre-exposition	3,909	0.7
Released from treatment	10,291	1.9
Observation	239,510	44.4
Observation + Vaccine	127,212	23.6
Vaccine	111,427	20.7
Serum + Vaccine	25,890	4.8
Re-exposition	1,791	0.3
No data	18,945	3.5
**Performed Treatment**		
**Anti-rabies vaccine**	**538,975**	**100.0**
Yes	240,627	44.6
No	298,348	55.4
**Doses given**	**240,627**	**100.0**
1 dose	44,258	18.4
2 doses	89,755	37.3
3 doses	28,941	12.0
4 doses	26,154	10.9
5 doses	51,519	21.4
**Anti-rabies serum doses given**	**538,975**	**100.0**
Yes	22,122	4.1
No	183,364	34.0
Unknown	8,591	1.6
No data	324,898	60.3
**Infiltration at the wound site**	**538,975**	**100.0**
Total	1,002	0.2
Partial	2,497	0.5
Not used	10,331	1.9
No data	525,145	97.4

Source: Author, 2024

Considering the characteristics of the notified aggressions, the type of exposure was classified as indirect contact in 0.9% (5,057/538,975) of the cases, minor accident in 34.3% (184,736/538,975), and severe accident in 61.4% (330,906/538,975). In Araçatuba, SP, a study that considered a 21-year period (1990-2010) identified a higher frequency of minor accidents (76.4%)^14^. When classification of the exposure characteristics was not possible owing to a lack of information (0.9%; 4,722/538,975) or incomplete data (2.5%; 13,554/538,975), the notifications were disregarded for further analyses.

The vaccine was indicated in 49.4% (266,320/538,975) of the anti-rabies medical care cases (Table 1). However, the doses were effectively administered to 44.6% of the victims, most frequently the 2-dose (37.3%) followed by the 5-dose (21.4%) protocol. The data show that 41.3% (99,353/240,627) of the vaccinated individuals (Table 1) were administered a number of doses (1, 3, or 4 doses) different from those recommended by the Ministry of Health PEP guidelines^5^ ^,^ ^16^, resulting in wasted public resources and unnecessary exposure to the vaccine. This was also observed in the city of Araçatuba^14^ and in other states of Brazil in 2017, when vaccines were inadequately administered in 37% of the cases, whereas serum and/or vaccine were underutilized for high-risk patients and were also unnecessarily administered to 8% of individuals^18^. 

Of the victims who were vaccinated, 26.3% (63,204/240,627) had their treatment interrupted, 38.9% of them following the recommendation of the local health unit or because of transference, and 61.1% abandoned the treatment, of whom 87.5% (33,797/38,627) were contacted by the health unit to resume treatment. In a period similar to our study (2010 and 2015), the interruption rates studied in Rio de Janeiro and São Paulo were quite similar (24.2% and 26.3%, respectively), with 89.3% of victims abandoning treatment^31^. In Brazil, from 2014 to 2019^11^ the results were similar to ours, with 24,3% of treatment interruptions, most of them due to abandonment (62,7%), from which, in 77.1% of the cases, the victims were contacted by the health unit. The treatment interruption rate is higher in São Paulo than in the whole country; however, the health services of São Paulo are more proactive towards seeking and contacting victims who abandoned treatment.

Anti-rabies serum was indicated in 4.8% of the cases in our study but was effectively administered in only 4.1% of the notification cases. Information regarding serum infiltration into wounds was also inconsistent, as only 0.7% of notifications had this record (Table 1). Data from Brazil (2009-2013) revealed that both serum and vaccine were indicated in 7.9% of the cases but effectively administered in 6.6%^29^, while in Rio de Janeiro, serum was indicated in 16.1% of cases, with no data available on the doses effectively administered^31^.

The data inconsistency in some fields of the form suggests a lack of monitoring by professionals handling the forms after the initial anti-rabies care. In three Brazilian states, healthcare professionals were interviewed to identify errors in the SINAN data and observed a relatively limited amount of time to complete the form, a large number of fields, errors when digiting paper forms, and a lack of feedback on the errors detected^32^. Given the high risk of contracting rabies due to inadequate prophylaxis, the data registered in the notification form should be evaluated by trained professionals to follow the guidelines required by the MS^5^ ^,^ ^16^, and transcription to SINAN should only be performed with robust data, or at least with evidence of active search.

Most of the treatments (71.9%; 357,6^2^3/497,069) indicated followed the recommendations of the MS^5^ ^,^ ^16^ (Table 2), with a higher frequency of appropriate conduct for severe accidents (75.1%; 237,556/316,287), followed by minor accidents (67.0%; 117,988/176,044). Furthermore, after monitoring the animals during the observation period, the prophylaxis indicated was deemed appropriate in the majority of cases (70.1%; 245,271/350,137). The high frequency of appropriately recommended PEP conducted in the state of SP is similar to that in other states such as Paraná (58.4%)^25^, but lower than the values observed in Porto Alegre/RS (96.2%)^33^ and Ceará (95.8%)^26^. Data from the whole country showed that in 89.3% of the minor accidents, the conduct was adequate/appropriate, and for severe accidents, in 51.4% of the cases, it was adequate^11^, which is the inverse of our observed results. As in SP state, the increase observed in the overall PEP use until 2015 in Brazil points to the underuse of vaccines or serum as the main cause of inappropriate PEP, that is, when they are required for high-risk (severe) bites. In 2015, when Brazil experienced a vaccine shortage, a reduction in PEP use occurred for the following two years, which also reduced the appropriate treatments by 45% in 2017 and consequently increased underuse by 12% from 2015 to 2017^18^.

**TABLE 2:** Classification of conduct and treatment in human anti-rabies care, as recommended by the Ministry of Health in the state of São Paulo, Brazil, between 2013 and 2017.

TYPE OF EXPOSURE	EXECUTION						Total
	Adequate		Inadequate		Inconclusive		
	N°	%	N°	%	N°	%	
**Prescribed conduct**							
Indirect contact	2,079	41.1	2,659	52.6	319	6.3	5,057
Minor accident	117,988	63.9	58,056	31.4	8,692	4.7	184,736
Severe accident	237,556	71.8	78,731	23.8	14,619	4.4	330,906
Total	357,623	68.7	139,446	26.8	23,630	4.5	520,699
**Performed treatment**							
Indirect contact	2,623	51.9	2,434	48.1	-	0.0	5,057
Minor accident	82,446	44.6	39,084	21.2	63,206	34.2	184,736
Severe accident	160,202	48.4	63,348	19.1	107,356	32.4	330,906
Total	245,271	47.1	104,866	20.1	170,562	32.8	520,699

**Source:** Author, 2024.

Among individuals exposed only to indirect contact with animals, the prevalence of inappropriate conduct was higher (56.1%; 2,659/4,738); however, when evaluating the post-animal observation period, 51.9% (2,623/5,057) were considered adequate, with the individuals being released from treatment. Indirect contact with an animal, designated by the WHO as category I^15^ exposure, is not considered a risky accident, and the use of immunobiologicals is unnecessary regardless of animal condition^5^ ^,^ ^16^. In cases of minor (category II) or severe (category III) accidents, prophylactic treatment must be provided to the victim according to the characteristics of the aggressor. It is worth emphasizing that in all cases where the possibility of exposure to rabies virus is present, immediate cleansing with soap and water is essential^5^ ^,^ ^15^ ^,^ ^16^. 

Considering that only 520,699 forms contained data that allowed the identification of the type of exposure, the treatment indicated was evaluated in 95.5% of the notifications. However, owing to the inconsistent information observed in the SINAN database, the recommended conduct and treatments performed were classified in 67.2% of anti-rabies care cases, while 32.8% remained inconclusive (Table 2). These inconsistencies or the lack of information in the forms could also be attributed to several knowledge gaps among health professionals assessing bite patients in Brazil, from evaluating the dog’s health condition to selecting the appropriate PEP regimen^32^. 

**FIGURE 2:**
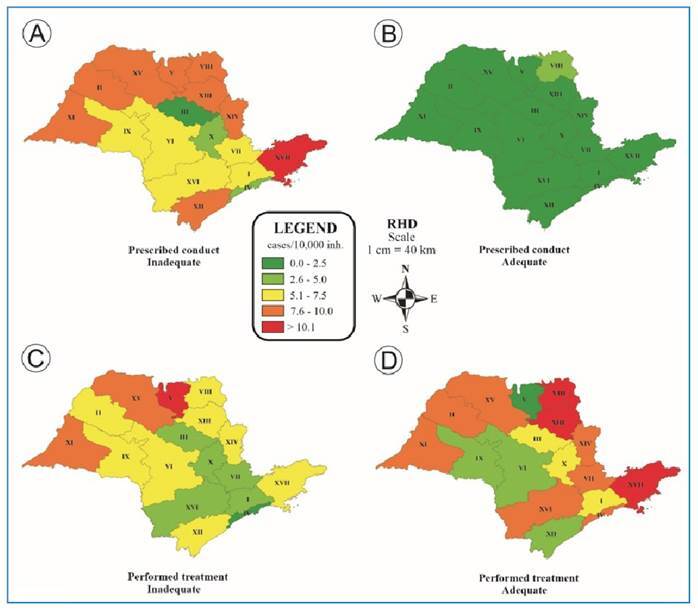
Distribution of the coefficient of incidence of the prescribed conducts and treatments performed in human anti-rabies care according to the Regional Health Departments (RHD I to XVII) in the state of São Paulo, Brazil, from 2013 to 2017.

The occurrence of inadequately prescribed conduct was greater than 7.5/10,000 inhabitants in 52.9% of the RHD (Figure 2A). Furthermore, the fields of the notification form were adequately filled out during the initial care period, resulting in a low rate (<2.6/10,000 inhabitants) of inconclusive conduct (Figure 2B). When evaluating individuals regarding the treatment performed, only three RHD had a rate greater than 7.5/10,000 inhabitants (Figure 2C); however, 58.8% of the RHD had inconclusive notifications (Figure 2D). These values are much lower than those observed in the state of Ceará, where more than 90% of individuals receive inadequate treatment, with an incidence of 120-225 cases per 10,000 inhabitants in some municipalities^26^, reinforcing once again the importance of correctly filling out the forms^32^.

*Prescribed conducts were classified as inappropriate **(A)** and inconclusive **(B)**, and performed treatments were classified as inappropriate **(C)** and inconclusive **(D)**. Source: Author, 2024.

The prescribed conduct and the treatment received were considered appropriate; that is, they followed the prophylaxis standards of the Brazilian MS^5^ ^,^ ^16^ in only 44% of the cases, while 66% corresponded to inadequate and inconclusive ones (Table 3). We observed that the chance of the treatment being properly administered was higher when the treatment was appropriately prescribed (odds ratio [OR], 16.1; 95% CI 15.9 - 16.4). Nevertheless, inadequate treatments resulted in 246,787 doses of vaccine and 8,888 doses of anti-rabies serum being administered, disregarding the MS recommendations^5^ ^,^ ^16^.

**TABLE 3:** Relationship between the number of prescribed conduct and performed treatment for human anti-rabies care according to the recommendation of the Ministry of Health in the state of São Paulo, Brazil, from 2013 to 2017.

	PERFORMED TREATMENT							
PRESCRIBED CONDUCT	Adequate		Inadequate		Inconclusive		**Total**	
	N°	%	N°	%	N°	%	N°	%
Adequate	229,058	44.0	37,243	7.2	91,322	17.5	357,623	68.7
Inadequate	11,133	2.1	65,791	12.6	62,522	12.0	139,446	26.8
Inconclusive	5,080	1.0	1,832	0.4	16,718	3.2	23,630	4.5
Total	245,271	47.1	104,866	20.1	170,562	32.8	520,699	100.0

**Source:** Author, 2024.

The total number of inappropriately administered immunobiological agents may be even higher, considering that 252,680 and 7,252 doses of the vaccine and antirabies serum, respectively, were administered after inconclusive and unknown (no information) visits. The inappropriate use of immunobiologicals is similar in other regions such as Paraná^24^, where 28.1% and 13.4% of dispensed treatments were considered insufficient and excessive, respectively; in Belo Horizonte/MG^34^, 22.1% and 11.5% of the individuals received excessive and insufficient treatment, respectively; and in Cuité/PB^28^, 3.3% were administered excessive doses.

The evaluation of the prescribed conduct and the treatment performed after the animal’s observation period, considering the PEP recommendations^5^ ^,^ ^16^, showed that in both cases, animal observation was the most frequently conducted, with a frequency of 90.9% (217,710/239,510) and 67.8% (162,291/239,510), respectively. Notifications recommending vaccine only (44.2%; 49,279/111,427) and serum + vaccine (34.4%; 8,917/25,890) had the highest frequency of inconclusive interpretation because of either a lack of or conflicting information in the records, differing from the data reported in the state of Paraná (80%)^25^ and in the municipality of Belo Horizonte/MG (46.2%)^34^, where observation + vaccine was the most appropriate indication.

## CONCLUSION

From 2013 to 2017, the occurrence of human antirabies care attendance in the state of SP was similar to that in Brazil as a whole. Severe accidents were the most frequent; therefore, animal observation for 10 days was the most indicated conduct. The suggested prophylactic conduct mostly followed the recommendations of the Ministry of Health for post-exposure human rabies prophylaxis; however, the use of immunobiologicals was considered excessive. Furthermore, many notifications with incomplete or inadequate information were observed.

Steps towards improving the data collection process and training programs for professionals entering the data into the forms are suggested. Information technology is a powerful ally, and with adequate software parameterization, it becomes easy to detect conduct diverging from that recommended by the MS, enabling proactive actions regarding the most appropriate treatment, whether in the inclusion or suspension of immunobiological doses. 
